# Community assembly and functional leaf traits mediate precipitation use efficiency of alpine grasslands along environmental gradients on the Tibetan Plateau

**DOI:** 10.7717/peerj.2680

**Published:** 2016-11-08

**Authors:** Shaowei Li, Jianshuang Wu

**Affiliations:** 1 Lhasa National Ecological Research Station, Key Laboratory of Ecosystem Network Observation and Modelling, Institute of Geographic Sciences and Natural Resources Research, Chinese Academy of Sciences, Beijing, China; 2 Functional Biodiversity, Dahlem Center of Plant Sciences, Free University of Berlin, Berlin, Germany

**Keywords:** Leaf functional traits, Community species assembly, Carbon isotope composition, Rain use efficiency, Tibetan alpine grasslands, Regional precipitation gradients

## Abstract

The alpine grasslands on the Tibetan Plateau are sensitive and vulnerable to climate change. However, it is still unknown how precipitation use efficiency (PUE), the ratio of aboveground net primary productivity (ANPP) to precipitation, is related to community assembly of plant species, functional groups or traits for the Tibetan alpine grasslands along actual environmental gradients. We conducted a multi-site field survey at grazing-excluded pastures across meadow, steppe and desert-steppe to measure aboveground biomass (AGB) in August, 2010. We used species richness (SR), the Shannon diversity index, and cover-weighted functional group composition (FGC) of 1-xerophytes, 2-mesophytes, and 3-hygrophytes to describe community assembly at the species level; and chose community-level leaf area index (LAI_c_), specific leaf area (SLA_c_), and species-mixed foliar δ^13^C to quantify community assembly at the functional trait level. Our results showed that PUE decreased with increasing accumulated active temperatures (AccT) when daily temperature average is higher than 5 °C, but increased with increasing climatic moisture index (CMI), which was demined as the ratio of growing season precipitation (GSP) to AccT. We also found that PUE increased with increasing SR, the Shannon diversity index, FGC and LAI_c_, decreased with increasing foliar δ^13^C, and had no relation with SLA_c_ at the regional scale. Neither soil total nitrogen (STN) nor organic carbon has no influence on PUE at the regional scale. The community assembly of the Shannon index, LAI_c_ and SLA_c_ together accounted for 46.3% of variance in PUE, whilst CMI accounted for 47.9% of variance in PUE at the regional scale. This implies that community structural properties and plant functional traits can mediate the sensitivity of alpine grassland productivity in response to climate change. Thus, a long-term observation on community structural and functional changes is recommended for better understanding the response of alpine ecosystems to regional climate change on the Tibetan Plateau.

## Introduction

Precipitation determines spatial and temporal variability in productivity of diverse temperate and alpine grasslands worldwide (Bai et al., 2008; Knapp & Smith, 2001; Sala et al., 2012; Sala et al., 1988; Shi et al., 2014; Yang et al., 2008). It is important to elucidate the mechanisms underlying the relationships between productivity, plant diversity, and precipitation with ongoing global change (Fay et al., 2011; Huxman et al., 2004; Knapp et al., 2008; Knapp et al., 2002; Shen et al., 2015; Weltzin et al., 2003). That is because changing precipitation regimes and other anthropogenic disturbances likely alter community composition and ecosystem functionality, and consequently affect ecosystem services and human welfare (Carmona et al., 2012; Christensen et al., 2004; Fay et al., 2011; Fay et al., 2008; Fernandez-Going, Anacker & Harrison, 2012; Osem, Perevolotsky & Kigel, 2002; Varnamkhasti et al., 1995).

Indeed, aboveground net primary productivity (ANPP), a key integrative ecosystem function, has been well documented to increase across vegetation types with increasing annual or seasonal precipitation gradient (Hu et al., 2007; Huxman et al., 2004; Jiang et al., 2015; Knapp & Smith, 2001; Sala et al., 1988; Yang et al., 2008; Yang et al., 2009). A large number of studies have accepted the term of precipitation use efficiency (PUE, the ratio of ANPP to precipitation) as a proxy of the sensitivity of productivity response to precipitation, because it has normalized the effect of water availability by placing productivity on a per-unit-of-precipitation basis (Bai et al., 2008; Gao et al., 2011; Hu et al., 2010; Huxman et al., 2004; Le Houerou, 1984; Paruelo et al., 1999; Varnamkhasti et al., 1995; Vermeire, Heitschmidt & Rinella, 2009; Yang et al., 2010). Most previous studies focused to examine the manner of productivity in response to changes in precipitation regimes (Bai et al., 2008; Gao et al., 2011; Hu et al., 2007; Paruelo et al., 1999; Sala et al., 1988; Yang et al., 2010); however, rare research has examined the potential and inherent regulating mechanisms of community assembly on ecosystem productivity.

As reported by Paruelo et al. (1999), differences in PUE among biomes can be attributed primarily to vegetation constraints under the given biogeochemical background. For example, O’Connor, Haines & Snyman (2001) and Knapp et al. (2002) found that PUE was influenced mostly by community composition and that species complementarity ensured greater and more stable productivity in species rich grasslands. In the American mixed-grass prairies, Vermeire, Heitschmidt & Rinella (2009) also found that PUE was likely more responsive to community composition of plant functional groups than to amount and seasonal distribution of precipitation. In the temperate sandy grasslands of Inner Mongolia, China, Zuo et al. (2012) even confirmed that productivity was driven indirectly by community composition, which was determined by or co-evolved with abiotic environmental variables. However, the literature on PUE across different alpine grasslands on the Tibetan Plateau, especially focusing on the mechanistic understanding of the effects of species assembly and/or functional traits, is relatively limited.

Relative growth rates and water use strategies that generally differ among species are likely regulated by both plant morphological and physiological traits. Foliar functional traits, such as specific leaf area (SLA, leaf area per unit dry mass), stable carbon isotopic composition (*δ*^13^C, ^13^C/^12^C isotope discrimination) and leaf area index (LAI, leaf area per unit land area), are often used to examine the response of productivity to precipitation in grassland studies (Fernandez-Going, Anacker & Harrison, 2012; Hu et al., 2008; Luo et al., 2004; Orwin et al., 2010; Violle et al., 2007; Wei et al., 2011; Wellstein et al., 2011). For example, Hu et al. (2008) reported that shifts of community composition induced by climate change, and consequent changes in community-level LAI, can significantly affect water and carbon cycles in temperate grasslands. A lot of studies found that SLA can serve as a good predictor of plant strategies on resource capture, usage and availability (Poorter & Evans, 1998; Wilson, Thompson & Hodgson, 1999). It is a prevailing view that *δ*^13^C can be used as an indicator of plant intrinsic water use efficiency, because it relates to leaf stomatal behavior in photosynthesis (Caldeira et al., 2001; Flanagan & Farquhar, 2014; Seibt et al., 2008; Song et al., 2008). Therefore, any change in community species assembly or plant functional traits can consequently mediate the response of grassland productivity to climate change at a given temporal and spatial scale (Reich et al., 2003; Wang et al., 2012).

Alpine grasslands on the Tibetan Plateau have increasingly gained attention for their sensitivity and vulnerability to climate change and overgrazing because severe habitat conditions constrain species to recruit from small local species pool there. Considerable studies have examined impacts of climate change and grazing disturbance on species diversity (Chen et al., 2008; Wu et al., 2012), above- and below-ground biomass (Li et al., 2011; Sun, Cheng & Li, 2013; Yang et al., 2009; Zeng, Wu & Zhang, 2015), and the plant diversity-productivity relationship (Ma et al., 2010; Wang et al., 2013; Wu, Shen & Zhang, 2014). On this plateau, water stress is one of the main factors constraining productivity of alpine grasslands (Shen et al., 2015; Shi et al., 2014). Therefore, determining how plants use precipitation may help understanding the underlying mechanisms associated with the plant diversity-productivity relationship. However, to our knowledge, only Hu et al. (2010) and Yang et al. (2010) have recently documented the spatial PUE pattern across diverse alpine grasslands, reporting a linear PUE-MAP (mean annual precipitation) relationship and a hump-shaped PUE-MAP relationship, respectively, along the precipitation gradient across the Qinghai-Tibetan Plateau. Indeed, both studies provided valuable examinations of soil nutrients, species richness (SR) and canopy coverage in controlling the spatial variation in PUE, however, they did not clarify whether and how community intrinsic structural properties, such as species composition and plant traits assembly, can also affect PUE of the Tibetan alpine grasslands. To better manage alpine pastures, it is necessary to estimate the relative contribution of climatic conditions, soil nutrients and vegetation regimes to the variability in response of alpine grassland productivity to precipitation.

In this study, we conducted a multi-site transect survey across the northern Tibetan Plateau in summer 2010, with maximum aboveground biomass (AGB), species diversity indices and community leaf traits measured, and climate and soil variables collected. Here, we had three objectives for the analyses of PUE variations across alpine grassland types at the regional level. The first is to clarify the spatial variation of PUE along climatic and soil nutrient gradients across zonal alpine grasslands on the northern Tibetan Plateau. In previous studies, PUE was often regressed against mean annual or seasonal precipitation. This did not make sense because PUE was also derived from precipitation. In this study, in addition to soil influences, we mainly focused on the independent effect of temperature alone, and the combined effect of both temperature and precipitation on PUE at the community level. Our second objective is to detect whether and how species composition and leaf functional traits can influence the spatial variation in PUE along climatic and resource gradients across the northern Tibetan Plateau. Finally, the third objective is to disentangle the relative contribution of climate, soil, and community properties to PUE across alpine grasslands on the northern Tibetan Plateau.

## Materials and Methods

### Study area

Field surveys were conducted at alpine grasslands across the northern Tibetan Plateau (4653–4910 m, 31.38–32.88°N, 79.78–91.96°E) in August, 2010. This area is considered as an ideal study platform for alpine grassland ecology research because of the evident environmental gradients longitudinally extending across the northwestern hinterlands of the Qinghai-Tibetan Plateau (Li et al., 2011; Wu et al., 2013b). The mean annual temperature and precipitation along this transect vary from −2.55 °C and 462.9 mm in the most-eastern site, respectively, to 0.9 °C and 66.5 mm in the most-western site (Wu, Shen & Zhang, 2014). In this region, most plants usually begin to green in May and to senesce in September, with approximately 85% of annual rainfall occurring during that period. The longitudinal zonation of alpine grasslands is consistent with climatic constraints, with a westward progression from semi-humid alpine meadow (AM dominated by *Kobresia pygmaea* and *K. humilis*) to semi-arid alpine steppe (AS dominated by *Stipa purpurea* and *Carex ivanovae*) and to arid alpine desert-steppe (ADS dominated by *S. glareosa*, *S. breviflora* and *Oxytropis chiliophylla*) (Li et al., 2011; Wu, Shen & Zhang, 2014).

### Sampling design

**Figure 1:**
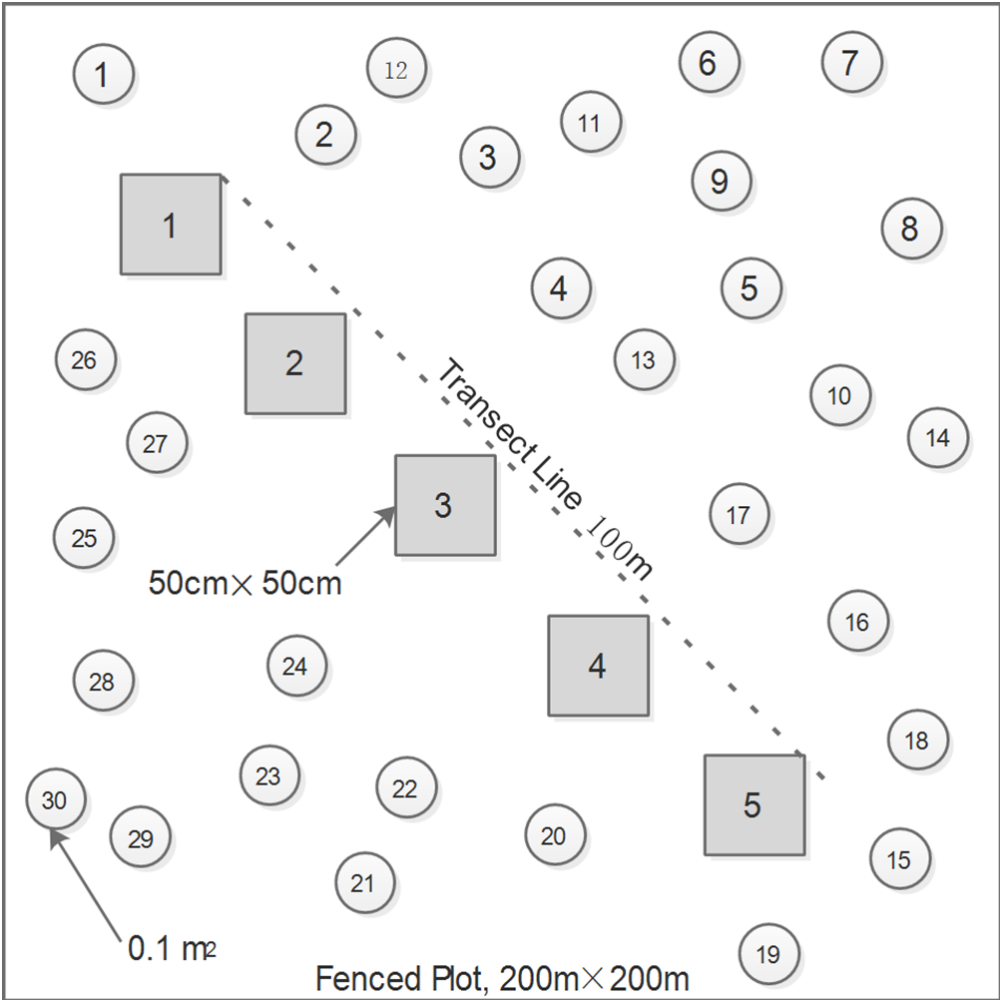
The layouts of one 100-m transect line, five 50 × 50 cm quadrats, and thirty 0.1-m^2^ circles randomly distributed within each plot.

To minimize the influences of complicating factors (e.g. livestock grazing and fencing duration) on measurements of species diversity, biomass, and leaf traits, we rigorously restricted our surveys to the 15 sites excluded from livestock grazing since 2006, five sites for each grassland type, to cover the represent natural communities of the three zonal alpine grassland types. Generally, the grazing exclosures were greater than 20 hectares in size. These ungrazed pastures were separated at intervals of approximately 50–80 km from each other across the northern Tibetan Plateau. At each site, we firstly set up a 200 × 200 m plot, approximately 200 m away from the exclosure edges, and then placed five 50 × 50 cm quadrats systematically at equal intervals along a 100-m transect line that was randomly laid out within the plot (Fig. 1). Species height (cm) and percent cover (%) of all the plants present at each quadrat were recorded before harvesting the living biomass. We measured the height of three individuals at least per species, thus an average height was used for further calculations. Plant biomass was cut to soil surface, sorted by species, and dried at 65 °C for 48 h in an oven prior to weighing. Most plants in alpine grasslands on this plateau generally reach their maximum coverage in August (Ma et al., 2010; Shi et al., 2014). Therefore, the maximum AGB can be viewed as ANPP, and the ratio of AGB to precipitation can be accepted as PUE in this study (Yang et al., 2010). In addition, thirty 0.1-m^2^ circles were randomly placed within each plot to determine the local species pool and species relative frequency (Fig. 1).

### Species composition

Based on the quadrat data, community assembly was determined by three biodiversity indices, SR, the Shannon diversity index, and plant functional group composition (FGC). SR was defined as the number of species occurring in each quadrat. Here, we used the sampling-circle data to determine species relative frequency (*F_r_*), which was estimated as the ratio of the occurrences (*N_i_*) of a given species to the total occurrences of all the species recorded in the thirty sampling circles. Species relative coverage (*C_r_*) was defined as the ratio of the absolute coverage of a given species (*C_i_*_)_ to the cover sum of all the species within a given quadrat. Meanwhile, species relative height (*H_r_*) was determined as the ratio of the absolute height of a given species (*Hi*) to the height sum of all the species within a given quadrat. Thus, we calculated the Shannon diversity index (*H*) from the relative importance value (*P*_*i*_) as the following equations:

(1) $${P_i} = ({C_r} + {H_r} + {F_r})/3$$

(2) $$H = - \sum\limits_{i = 1}^n {{P_i}ln{P_i}} $$

A few recent studies have indicated that FGC discriminated by differential water ecological strategies likely regulates the productivity of alpine grassland communities in responding to climatic fluctuation and grazing disturbance on the Qinghai-Tibetan Plateau (Song et al., 2008; Wu et al., 2013a; Zhou et al., 2011). Therefore, we referred to the Flora of Tibet (Wu, 1987) for descriptions about water use strategies-xerophytes, mesophytes, or hygrophytes-for all the species sampled in this study. We categorized and provisionally scored plants into the three plant functional groups, 1-xerophytes, 2-mesophytes, and 3-hygrophytes. Different plant functional groups might have differential root and leaf properties that affect their sensitivity and fitness in response to precipitation, such as root distribution and leaf density. Compared to forbs and other woody plants, for example, grasses and herbaceous plants are relatively shallow-rooted and primarily rely on near-surface soil water and nutrients (Chen, Bai & Han, 2003; Ogle & Reynolds, 2004; Wu et al., 2013b). Finally, we calculated plant FGC by a coverage-weighted approach as follows:

(3) $${\rm FGC}\,{\rm{ = }}\sum\limits_{i = 1}^n {PF{G_i}{\rm{ }} \times {\rm{ }}Co{v_i}} $$

where *PFG*_*i*_ is the provisional score of the given species *i* according to its water ecological strategy, *Cov*_*i*_ is the coverage of species *i*, and *n* is SR within the sampled quadrat. Thus, a higher value of FGC means relative more hygrophytes and mesophytes than xerophytes.

### Leaf functional traits

The climate change-induced shifts in vegetation structure, and consequently in leaf functional traits, such as LAI and SLA, likely affected both water and carbon cycles of grassland communities (Hu et al., 2008). In this study, we scanned 20–30 fresh mature leaves for each species at our plots using a leaf area meter (AM200; ADC Bio-Scientific Limited, Herts, UK). The species-specific leaf area (SLA_s_) was defined as the ratio of fresh leaf area to their dry weight. We partitioned leaves by species from the entire AGB to calculate leaf mass fraction (LMF_s_) for each species occurring at our sites. Finally, using LMF_s_ as a scaling factor, we scaled from the individual species level (LAI_s_ and SLA_s_) up to the community level (LAI_c_ and SLA_c_):

(4) $${\rm{LA}}{{\rm{I}}_{\rm{c}}} = {\rm{}}\mathop \sum \limits_{i = 1}^n ({\rm{SL}}{{\rm{A}}_{{\rm{si}}}}{\rm{}} \times {\rm{LM}}{{\rm{F}}_{{\rm{si}}}} \times {\rm{AG}}{{\rm{B}}_{{\rm{si}}}})$$

(5) $${\rm{SL}}{{\rm{A}}_{\rm{c}}} = {\rm{LA}}{{\rm{I}}_{\rm{c}}}/\mathop \sum \limits_{i = 1}^n ({\rm{LM}}{{\rm{F}}_{{\rm{si}}}} \times {\rm{AG}}{{\rm{B}}_{{\rm{si}}}})$$

where *i* and *n* are the *i*th species and SR in each quadrat, respectively.

Foliar stable carbon isotopic composition (δ^13^C) is another important physiological indicator that has been used to estimate plant intrinsic water use efficiency at both species and community levels (Caldeira et al., 2001; Farquhar, Ehleringer & Hubick, 1989; Flanagan & Farquhar, 2014; Lamont, Groom & Cowling, 2002; Seibt et al., 2008; Song et al., 2008). It is expensive to do isotopic analyses of plant stable carbon isotope composition in China. For this reason, we did not analyze stable carbon isotopic composition for each species. Instead, we analyzed the mixed foliar materials at quadrats in which we measured SR. These species-mixed foliar samples and leaves of a few dominant species were washed with distilled water, oven-dried at 65 °C for 48 h and then ball milled to be fine homogeneous powder for further analyses. ^13^C/^12^C ratios were determined by an isotopic mass spectrometer (Thermo MAT253; Thermo, Bremen, Germany). All isotopic analyses were performed at the central physicochemical laboratory of the Institute of Geographic Sciences and Natural Resources Research, Chinese Academy of Sciences. Stable carbon isotopic values were calculated with Eq. (6):

(6) $${\delta ^{13}}C = \left[ {({R_{{\rm{sample}}}} - {R_{{\rm{standard}}}})/{R_{{\rm{standard}}}}} \right] \times {\rm{1000}}$$

where *R*_sample_ and *R*_standard_ represent the abundance ratio of ^13^C/^12^C in the sample and the standard, respectively. The universally accepted standard of Pee Dee Belemnite (PDB) was used in the isotopic analyses.

### Climate data and soil nutrients

Precipitation outside plant growing season (May–September) is rare and unavailable for plant growth due to low-temperature stress and gale-force winds in cold months (Yu, Wang & Wang, 2010). Therefore, we defined PUE as the ratio of maximum AGB to growing season precipitation (GSP) in this study, rather than to MAP as did Yang et al. (2010). We downloaded daily records of temperature and precipitation from May to September of the 39 meteorological stations in the Tibetan Autonomous Region, China, from the National Meteorological Information Centre (NMIC) of China Meteorological Administration (CMA). Daily mean temperatures that were greater than 5 °C (accumulated active temperature (AccT)) were summed to describe thermal conditions during plant growing months. In the following steps, we used ANUSPLIN version 4.3 (Hutchinson, 2004) to produce climatic raster surfaces of the three climatic variables (GSP, AccT and GSP/AccT) and then we extracted them to match site locations in ArcGIS 10.2 (ERSI, Redlands, CA, USA). GSP/AccT was used as a substitute of climate moisture index (Wang et al., 2013) to quantify the combined effect of temperature and precipitation. In this study, two topsoil nutrients, soil organic carbon (SOC) and soil total nitrogen (STN), were collected as potential explanatory variables from our previous studies (Li et al., 2011; Wu et al., 2013a; Wu et al., 2012).

### Data analysis

In this study, we firstly conducted multiple comparisons by one-way analysis of variance (ANOVA) with Turkey’s Honestly Significant Difference (HSD) test to examine differences in PUE and other explanatory variables among the three alpine grassland types, meadow, steppe, and desert-steppe across the northern Tibetan Plateau. In the second step, we plotted PUE against climatic, soil, and vegetation variables with ordinary least-squares (OLS) regressions to show the patterns of PUE along environmental gradients. Meanwhile, we also conducted Spearman correlation analysis to determine the covariations between climatic, soil and vegetation variables at the regional scale. Finally, we built a few general linear models (GLMs) with ANOVA to disentangle the relative contribution of climatic, edaphic and vegetation factors to PUE. In this step, to determine the optimal model, the least significant term was dropped until that the Akaike Information Criterion (AIC) value of the candidate model was the lowest and that all explanatory terms were significant. Meanwhile, we avoided including variable pairs with correlations of 0.5 or greater. We selected the optimal model by the AIC. Finally, the relative contribution of each explanatory variable to PUE was calculated as the percentage of variance explained (He et al., 2008; Ma et al., 2010). All statistical analyses were performed using the package” gmodels” in Rstudio (R Development Core Team, 2014), and figures were plotted in SigmaPlot 12.0 (Systat Software, NC., San Jose, CA, USA).

## Results

### Comparisons of climatic and soil variables among alpine grassland types

Considerable differences were observed in PUE and its potential abiotic explanatory variables across alpine grassland (one-way ANOVA with Turkey’s HSD test, Table 1). Mean PUE of AM sites was nearly 1.89 and 2.27 times, respectively, as high as that in both steppe and desert-steppe sites. GSP declined from 406.8 to 198.6 mm in ADSs. By contrast, AccT increased from 1,173 °C in AMs to 1,658 °C in ADSs. The contrary trends of temperature and precipitation across the northern Tibetan plateau made it more arid in ADSs than both in ASs and meadows, with mean climatic moisture index (CMI) 0.34 mm °C^−1^ (meadow) > 0.27 mm °C^−1^ (steppe) > 0.12 mm °C^−1^ (desert-steppe). Soils in AMs were also found to be more fertile with higher SOC content than soils in ASs and desert-steppes, however, no difference in STN was found among the three alpine grassland types.

**Table 1:** Mean values (ranges) for growing season precipitation (GSP, mm), accumulated temperature for daily values above 5 °C (AccT, °C), climatic moisture index (CMI, equaling to GSP/AccT, mm °C^−1^), topsoil organic carbon (SOC, %), soil total nitrogen (STN, %), species richness in the 50 × 50 cm quadrat, the Shannon diversity index (*H*), functional group composition (FGC, sum score of species water ecological strategies, 1-xerophytes, 2-mesophytes and 3-hygrophytes, weighted with plant group coverage), community leaf area index (LAI_c_, m^2^ m^−2^), community specific leaf area (SLA_c_, defined as leaf area per leaf mass, cm^2^ g^−1^), precipitation use efficiency (PUE, the ratio of peak AGB to GSP, g m^2^ mm^−1^), and species-mixed foliar stable carbon isotope composition determination (*δ*^13^C, ‰) within the zonal alpine grassland types: meadows, steppes and desert steppes. For *δ*^13^C, mean values of three sites per each grassland type were given due to the expensive cost of stable carbon isotope analysis. Different smaller letters indicate difference between alpine grasslands was significant at P < 0.05 (one-way analysis of variance with Turkey’s).

	Alpine meadows	Alpine steppes	Alpine desert steppes
GSP	406.8 (394.3–449.1) a	313.8 (267.9–380.3) b	198.6 (135.2–231.1) c
AccT	1173 (1092–1251) a	1200 (807–1515) a	1658 (1567–1771) b
CMI	0.34 (0.31–0.39) a	0.27 (0.19–0.40) a	0.12 (0.08–0.15) b
SOC	2.42 (0.82–4.14) a	1.37 (1.00–2.35) ab	0.78 (0.41–1.19) b
STN	0.13 (0.03–0.35) a	0.07 (0.05–0.11) a	0.06 (0.03–0.12) a
SR	11.4 (5.8–15.6) a	5.6 (2.0–10.8) b	4.4 (2.2–6.2) b
*H*	2.19 (1.63–2.51) a	1.37 (0.40–2.15) ab	1.16 (0.42–1.58) b
FGC	1.13 (0.43–1.72) a	0.15 (0.05–0.21) b	0.10 (0.08–0.13) b
LAI_c_	0.81 (0.60–1.18) a	0.29 (0.05–0.44) b	0.15 (0.11–0.21) b
SLA_c_	201.1 (165.9–249.2) a	171.1 (160.0–182.8) a	176.8 (168.1–183.7) a
PUE	0.123 (0.104–0.152) a	0.065 (0.013–0.114) b	0.054 (0.040–0.076) b
*δ*^13^C	−26.490	−25.996	−25.131

### Comparisons of community assembly of plant functional groups and traits

Community assembly of plant functional groups and traits also considerably differed among the three alpine grassland types (Table 1). Mean SR (11 species per 0.25 m^2^) at the plot level in AMs was 2.03 and 2.59 times, respectively, as high as that in ASs (5.6 species per 0.25 m^2^) and desert-steppes (4.4 species per 0.25 m^2^). The Shannon diversity index was nearly twice as high as that in ASs and desert-steppes. Mean plant FGC of 1-xerophytes, 2-mesophytes and 3-hygrophytes, weighted by absolute coverage, declined from 1.15 in AMs to 0.10 in alpine-steppes. LAI at the community level declined from 0.81 in AMs westwards to 0.15 in alpine-desert-steppes. SLA (cm^2^ leaf area g^−1^ leaf mass) declined from 201.1 in AMs to 171.1 in ASs, and then increased to 176.8 in ADSs. In general, no significant difference in community assembly of functional groups and traits was found between AS and desert-steppe communities, and AM had higher values than both steppe and desert-steppe.

### Spatial patterns of precipitation use efficiency along environmental gradients

**Figure 2:**
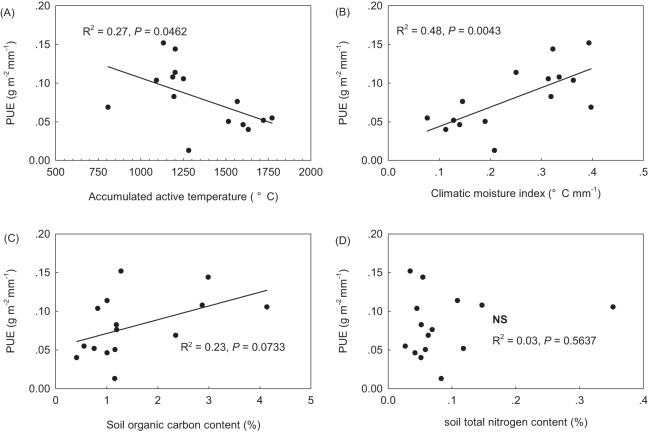
Precipitation use efficiency (PUE) along climate and soil gradients across the northern Tibetan Plateau. (A) accumulated active temperature when daily values are above 5 °C; (B) climatic moisture index (mm °C^−1^), equaling to the ratio of growing season precipitation to accumulated active temperature; (C) soil organic carbon in the topsoil (%); and (D) soil total nitrogen (%) in the topsoil.

PUE linearly decreased along the increasing AccT gradient, but linearly increased with increasing CMI (Figs. 2A and 2B). AccT and CMI were found to be negatively correlated with each other, and to be negatively and positively, respectively, correlated with GSP across sites (Table 2). PUE was found to marginally linearly increased with increasing SOC in the topsoil, but had no relation with STN (Figs. 2B and 2C). No correlation was found between SOC and STN across sites (Table 2).

**Table 2:** Pearson correlation coefficients between growing season precipitation (GSP), accumulated active temperature for daily values above 5 °C (AccT), climatic moisture index (CMI, equaling to GSP/AccT, mm °C^−1^), topsoil organic carbon (SOC, %), soil total nitrogen (STN, %), species richness in the 50 × 50 cm quadrat, the Shannon diversity index (*H*), functional group composition (FGC, sum score of species water ecological strategies, 1-xerophytes, 2-mesophytes and 3-hygrophytes, weighted with plant group coverage), community leaf area index (LAI_c_, m^2^ m^−2^) and community specific leaf area (SLA_c_, defined as leaf area per leaf mass, cm^2^ g^−1^) at 15 sites across the northern Tibetan Plateau.

	GSP	AccT	CMI	SOC	STN	SR	H	FGC	LAI
AccT	−0.868**								
CMI	0.914**	−0.975**							
SOC	0.668**	−0.543*	0.661**						
STN	0.075	−0.082	0.100	0.393					
SR	0.770**	−0.563*	0.570*	0.415	−0.150				
*H*	0.818**	−0.625*	0.629*	0.414	−0.129	0.974**			
FGC	0.864**	−0.704**	0.775**	0.679**	0.039	0.781**	0.804**		
LAI	0.900**	−0.704**	0.764**	0.689**	0.179	0.801**	0.839**	0.939**	
SLA	0.164	0.018	0.046	0.143	−0.229	0.218	0.132	0.186	0.129

**Notes:**

** correlation is significant at *p* < 0.01;

* correlation is significant at *p* < 0.05.

### Spatial patterns of precipitation use efficiency along community structure variables

**Figure 3:**
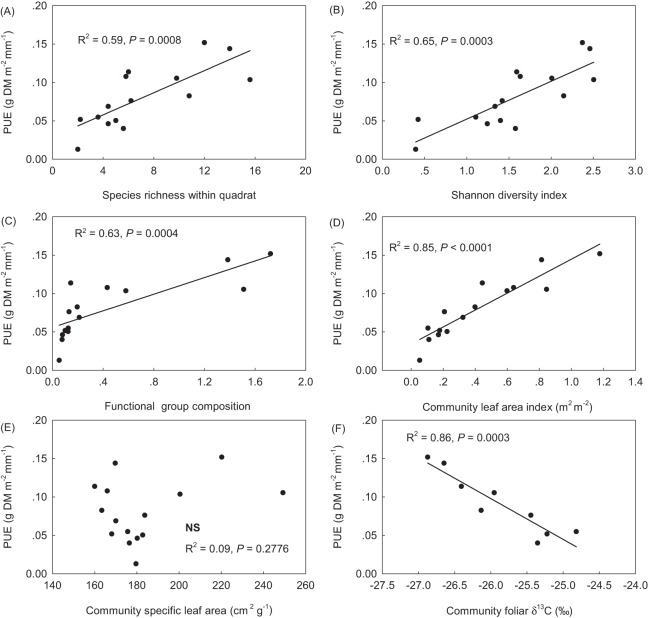
Relationships of precipitation use efficiency (PUE) to community composition of species (A & B), plant functional groups (C), and foliar functional traits (D–F). (A) species richness within the 50 × 50 cm quadrat; (B) the Shannon diversity index; (C) functional group composition, sum score of species water ecological strategies, 1-xerophytes, 2-mesophytes and 3-hygrophytes, weighted with plant group coverage; (D) community leaf area index (LAI_c_, m^2^ m^−2^); (E) community specific leaf area (SLA_c_, defined as leaf area per leaf mass, cm^2^ g^−1^), and (F) *δ*^13^C, the foliar stable carbon isotope composition.

**Figure 4:**
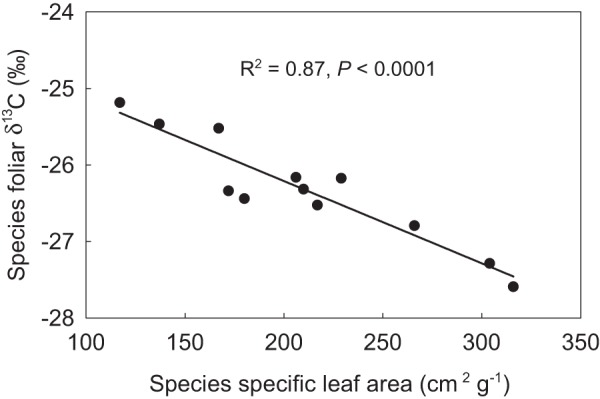
Relationship between specific leaf area (SLA) and foliar stable carbon isotope composition (*δ*^13^C) at the species level.

PUE linearly increased with SR, the Shannon diversity index, plant FGC, and community leaf area index (LAI_c_) (Figs. 3A–3D). PUE had no relation with community specific leaf area (SLA_c_) (Fig. 3E), but deceased with increasing foliar stable carbon isotope composition at the community level (Fig. 3F). Except of no correlation with STN, plant FGC and LAI_c_ were highly correlated with climatic variables and other vegetation properties (Table 2). Extreme correlations with absolute coefficient higher than 0.9 were found between GSP and CMI (0.914), between AccT and CMI (−0.975), between SR and the Shannon diversity index (0.974), and between plant FGC and LAI_c_ (0.939) (Table 2). Although SLA_c_ had no relation with climate variables, soil nutrients, or other community structural variables (Table 2), SLA was significantly related with foliar δ^13^C at the species level (Fig. 4).

### Partitioning the relative contribution of potential explanatory variables on PUE

When tested with the GLM, a strong climatic control was confirmed, with CMI explaining 47.9% of the total variance in PUE (Table 3, GLM2 with the lowest AIC and Bayesian Information Criteria (BIC) values). This was also visible in bivariate analysis with a linear regression of PUE against CMI (Fig. 2B). The community structural properties including the Shannon diversity index, LAI_c_ and SLA together accounted for 46.3% of the total variance in PUE. In GLM3, community leaf area accounted for 20.7% of the total variance in PUE, followed by SR (17.8%) and SLA_c_ (7.5%) (Table 3). When plant FGC was considered, community structural properties together explained 34.2 and 37.2% of the total variance in PUE, respectively (to see GLM1 and GLM4, Table 3). SOC and total nitrogen had no influence on PUE, and failed to enter the models (Fig. 2; Table 3).

**Table 3:** Summary of general linear models for the effects of climate, soil and community properties on precipitation use efficiency (PUE) across alpine grassland types on the northern Tibetan Plateau at the regional scale. Explanatory terms used included CMI, SR in the 50 × 50 cm quadrat, the Shannon diversity index (*H*), FGC (FGC, sum score of species water ecological strategies, 1-xerophytes, 2-mesophytes and 3-hygrophytes, weighted with plant group coverage), LAI_c_ (LAI_c_, m^2^ m^−2^), and SLA_c_ (SLA_c_, defined as leaf area per leaf mass, cm^2^ g^−1^). Because CMI was extremely correlated with GSP and AccT for daily values above 5 °C (AccT), with absolute spearman correlation coefficients higher than 0.9, GSP and AccT were excluded from the candidate GLMs. Two pairs of explanatory variables, SR vs. *H* (0.974) and FGC vs. LAI_c_ (0.939), extremely correlated with each other (Table 2), therefore, they were alternatively included into the four GLMs. d.f. degrees of freedom, M.S. mean squares, F variance ratio, P significance, %SS percentage of total sum of squares explained. Both AIC and BIC were provided for comparing model performance.

GLM1-Term	d.f.	MS	F	P	%SS	GLM2-Term	d.f.	MS	F	P	%SS
CMI	1	0.0106	26.65	< 0.001	47.8	CMI	1	0.0106	82.47	< 0.001	**47.9**
SR	1	0.0039	9.89	0.010	17.8	*H*	1	0.0049	38.43	< 0.001	22.3
FGC	1	0.0019	8.89	0.051	8.8	LAI	1	0.0038	29.83	< 0.001	17.3
SLA	1	0.017	4.26	0.067	7.6	SLA	1	0.0015	11.59	0.007	6.7
Residuals	10	0.0004			18.0	Residuals	10	0.0001			5.8
AIC	−69.01		BIC	−64.76		AIC	−85.95		BIC	−81.70	

GLM3-Term	d.f.	MS	F	P	%SS	GLM4-Term	d.f.	MS	F	P	%SS
CMI	1	0.0106	76.34	< 0.001	47.9	CMI	1	0.0106	32.01	< 0.001	47.9
SR	1	0.0039	28.32	< 0.001	17.8	*H*	1	0.0049	14.91	0.003	22.3
LAI	1	0.0046	32.95	< 0.001	20.7	FGC	1	0.0018	5.33	0.044	8.0
SLA	1	0.0016	11.90	0.006	7.5	SLA	1	0.0015	4.63	0.057	6.9
Residuals	10	0.001			6.3	Residuals	10	0.0003			14.9
AIC	−84.79		BIC	−80.54		AIC	−71.75		BIC	−67.51	

## Discussion

The data presented in this study illustrated that climate primarily constrains PUE of alpine grasslands on the Tibetan Plateau. Our results also indicated that vegetation structural properties including SR, community assembly, plant composition of different functional groups, and plant functional trait diversity can mediate the response of productivity to changing climate regimes. More importantly, we found that the explanatory variables that reflect community assembly of plant species, functional groups, and functional traits, can influence PUE as strongly as climatic variables. Therefore, we suggest that complex measures such as functional trait diversity, convergence and divergence of species niches, community assemblage of plant functional groups, are important predictors of ecosystem functioning with ongoing climate change for alpine grasslands.

Alpine grasslands have a long co-evolutionary history with severe abiotic environmental conditions on the Tibetan Plateau. Indeed, changes in precipitation regimes for the central Tibetan Plateau caused the spatial and temporal variation in grassland productivity and phenology (Shen et al., 2015; Shi et al., 2014). We accept the CMI to reflect the combining effect of precipitation and temperature together as did Wang et al. (2013). Our results showed that CMI alone explained 47.9% of the total variance in PUE across the sites in this study (Fig. 2; Table 3). Significant correlations of community structural and functional variables with CMI (Table 2) further confirmed that different PUE among alpine grassland types was the result of the environmental filtering effects on plant species composition and functional trait diversity. However, in our study, SOC and total nitrogen had no influence on PUE (Fig. 2), and both failed to enter the optimal GLM with multiple explanatory variables (Table 3). However, we cannot reject that that that loamy soils with higher organic carbon content in AM has a greater water-holding capacity that sandy soils in AS and desert-steppe (Yang et al., 2010). Knapp et al. (2002) reported that ANPP is more responsive to soil moisture variability than to mean soil water content. In addition, plant community composition and key carbon cycling process can be altered by increases in precipitation variability. A greater proportion of small rainfall events at arid desert-steppe may be more easily evaporated due to relatively sparser canopy structure, coarser soil texture and warmer soil temperature, whereas large rainfall events at the semi-humid meadow zone may be less evaporated and better hold up due to relative compacted canopy cover, finer soil texture and colder soil temperature (Table 1).

The alpine grasslands selected in this study cover a wide range of species diversity, productivity and environmental gradients, representing a good natural experiment platform from which to research climate-vegetation relationship on the Tibetan Plateau (Li et al., 2011). PUE for the three zonal alpine grassland types in our study (Table 1) were relatively lower than those reported in previous studies (Hu et al., 2010; Yang et al., 2010). For example, PUE for AMs and steppes reported by Hu et al. (2010) were nearly 2.7 times and twice the average values in our study. Although our sites cover a wide range in SR (from 2 to 16 species per 0.25 m^2^), average plant SR was lower when compared with AMs and steppes in the north-eastern and central Tibetan Plateau (Ma et al., 2010; Shi et al., 2014). It was reported that productivity was often greater and of lower annual variability for species-rich communities than species-poor ones across the Tibetan Plateau (Shi et al., 2014), possibly because the co-evolution of plant species assemblage with local climate condition and soil properties (Ma et al., 2010; Wang et al., 2013). Moreover, a positive linear relationship between productivity and SR has been reported in alpine grasslands on the Tibetan Plateau (Ma et al., 2010; Wang et al., 2013; Wu, Shen & Zhang, 2014). Our results also confirmed that SR, accounting for 17.8% of the total variance of PUE at the regional scale (GLM3 in Table 3), can be serve as a good predictor of PUE in this region (Fig. 3A). In the temperate sandy grasslands of Inner Mongolia, Zuo et al. (2012) argued that the positive correlation between plant diversity and productivity was indirectly driven by vegetation composition. The niche complementarity theory suggests that a species-rich community generally has more efficient resource utilization and more stable ecosystem functionality than species-poor communities (De Boeck et al., 2006; Marquard et al., 2009; O’Connor, Haines & Snyman, 2001). Therefore, the lower PUE in steppe and desert-steppe compared with that in AM in this study was likely caused by lower SR and the weaker complementary effect there.

In addition to SR, we also examined the effects of the Shannon diversity index and plant functional groups. A significant linear relationship was observed between PUE and the Shannon diversity index (Fig. 2B). Because of the significant correlation between the Shannon diversity index and SR in our case (Table 2), their relative contributions were found comparable to variability in PUE, accounting for 22.3 and 17.8% of the total variance of PUE by the Shannon diversity index in the optimal model and SR in the secondary optimal model (Fig. 3; Table 3). Therefore, our results supported the idea that that the productivity of alpine grasslands on the Tibetan Plateau is dependent not only on SR but also on species composition. The relative contribution of community composition of plant functional groups that have differentially evolved to adapt to serve habitat conditions on this plateau was not strong as expected (Table 3). PUE was found to increase with increasing the value of FGC (Fig. 3C), which reflects an overall water ecological strategy weighted by plant functional group cover at the community level. Communities dominated by mesophytic grasses have been found to respond more rapidly to precipitation change than communities that were dominated by xerophytic grasses (Paruelo et al., 1999). This is partly consistent with the finding of Cantarel, Bloor & Soussana (2013) that climate-induced decrease in above ground biomass may be driven by changes in the relative abundance of plant functional groups.

The current research on the relationship between biodiversity and ecosystem functionality has increasingly moved the focus from plant species to trait-based approaches (Orwin et al., 2010; Violle et al., 2007; Wellstein et al., 2011). In this study, we also found that PUE significantly increased with increasing LAI_c_ (Fig. 3), which explained 17.3% of the total variance. This was consistent with the positive linear relationship between PUE and vegetation cover observed in alpine grasslands by Hu et al. (2010), who suggested that climate change-induced shifts in vegetation structure, and consequently LAI may have a significant impact on the relationship between ecosystem carbon and water cycles in grasslands (Hu et al., 2008). Semi-arid grasslands are projected to be among the most sensitive ecosystems to changes in precipitation (Cherwin & Knapp, 2012). Life history and biogeochemical mechanisms can interact to influence the production response of alpine grasslands to precipitation. The evolutionary history and ecological attributes of species present in the vegetation assemblage can influence production potential as a result of constrains on growth rate imposed by trade-offs with traits for stress tolerance. Recruitment limitation can thus constrain local-scale SR and productivity, either by a lack of seeds or by reduced seedling growth, likely due to competition from the established vegetation (Zeiter, Stampfli & Newbery, 2006) The severe water stress due to less precipitation and warmer in AS and desert-steppe communities may not only lead to self-thinning but also to greater relative dominance of plant species that are more tolerant to drought with lower SLA and deeper roots(Wu et al., 2013b; Zhu et al., 2015). Foliar δ^13^C data in plant tissues has increasingly been used to infer intrinsic water use efficiency (Farquhar, Ehleringer & Hubick, 1989; Seibt et al., 2008). Both Song et al. (2008) and Zhou et al. (2011) also confirmed that the leaf δ^13^C of dominant species can reflect plant responses to the environmental water gradients on the Qinghai-Tibetan Plateau. In our results, the significant relationship of foliar δ^13^C to SLA at the species level (Fig. 3) did not result in a significant relationship between PUE and SLA_c_ (Fig. 2E). However, PUE was closely related to plant foliar δ^13^C and declined linearly with increasing community foliar δ^13^C (Fig. 2F). Therefore, we ascribed this outcome to uncertainties resulting from the assembly of species with differential water ecology strategies.

In summary, the productivity of alpine grassland communities across the northern Tibetan Plateau is controlled primarily by rainfall during the plant growing months. Our hypothesis that community structural properties and plant functional traits would mediate the sensitivity of alpine grassland productivity in response to precipitation change is supported, but the limited amount of data in this study cannot clarify the causal networks among climate change, community assembly and functional trait diversity. Our short-term study design does not allow us to answer the question of how ecosystem stability in the response of productivity to precipitation either. Clearly, long-term observation of community structural (and functional) changes in species and trait assembly is needed for predicting ecosystem response under future climatic scenarios, and for policy-making about sustainable management of alpine grasslands on the Tibetan Plateau.

## Supplemental Information

10.7717/peerj.2680/supp-1 Supplemental Information 1 Means of variables measured in this study for the fifteen sites.

## Additional Information and Declarations

### Data Deposition

The following information was supplied regarding data availability: The raw data has been supplied as Supplemental Dataset Files.
